# Modeling angle-resolved photoemission of graphene and black phosphorus nano structures

**DOI:** 10.1038/sdata.2016.31

**Published:** 2016-05-10

**Authors:** Sang Han Park, Soonnam Kwon

**Affiliations:** 1 Beamline Division Group of PAL-XFEL Project Headquarters, Pohang university of science and technology, 77 Cheongam-Ro, Nam-Gu, Pohang, Gyeongbuk 790-784, Korea

**Keywords:** Density functional theory, Characterization and analytical techniques, Method development

## Abstract

Angle-resolved photoemission spectroscopy (ARPES) data on electronic structure are difficult to interpret, because various factors such as atomic structure and experimental setup influence the quantum mechanical effects during the measurement. Therefore, we simulated ARPES of nano-sized molecules to corroborate the interpretation of experimental results. Applying the independent atomic-center approximation, we used density functional theory calculations and custom-made simulation code to compute photoelectron intensity in given experimental setups for every atomic orbital in poly-aromatic hydrocarbons of various size, and in a molecule of black phosphorus. The simulation results were validated by comparing them to experimental ARPES for highly-oriented pyrolytic graphite. This database provides the calculation method and every file used during the work flow.

## Background & Summary

The electronic structure of nano-scale materials determine their peculiar electrical and optical properties^1,2^. In particular, edge structures of nano-materials strongly affect their characteristics^3^. Therefore, the ability to correlate a specific structure to electronic properties is expected to enhance our understanding of nano-scale materials^4^.

The structure of nano-sized materials can be probed using various experimental tools such as transmission electron microscopy (TEM) and scanning transmission X-ray microscopy (STXM)^5,6^. Recently-developed nano-scale angle-resolved photoemission spectroscopy (nano-ARPES) is expected to probe both the structural and electronic properties of nano-scale materials simultaneously^7^.

Theoretically, the initial state can be calculated by density functional theory (DFT) and the initial state can be utilized to explain the ARPES results by using a plane-wave final-state approximation and Fourier transform formalism. However, this approximation disregards the complicated effects of the measurement process. To take into account these effects, a theoretical treatment that uses a one-step model to describe the photoemission process accurately has been developed. This method is an exact solution of photoemission from an atom. The final state is a continuum orbital; its radial part can be obtained by solving the Schrodinger equation, in which potential energy is the effective atomic potential^4,8,9^.

To generalize the theory to molecules, an independent atomic center (IAC) approximation has been developed^10,11^. The energy levels and wave functions of molecular orbitals are obtained using DFT calculations. The molecular orbitals are exploited to calculate the intensity of photoemission from the whole molecule. The photoemission from the whole molecule is assumed to be the sum of emissions from its constituent atoms. The present authors recently used this method to explain the ARPES simulation for nano-scale molecules^4^. By using this method, the effects of the measurement process, of the final state, and of polarization of exciting photons could be easily interpreted. Here, we present the raw data used in ref. 4, for use in further studies on nano-scale layered materials. We added a complete dataset that was not included in ref 4, and all intermediate data that can be used for pedagogical purposes.

## Methods

### Study design

Our dataset contains results of photoelectron intensity simulation using IAC of hexagonally-symmetric poly-aromatic hydrocarbon (PAH) molecules with zigzag- or armchair-edged structures, and of black phosphorus (BP). The PAHs studied have from 24 to 864 carbon atoms that are labeled following the indexing rule defined by Stein *et al.* ^12^ Using this calculation method, one can estimate ARPES result of molecules at every molecular orbital for appropriate experimental setup for measurements, such as photon energy, polarization, and geometry of experimental apparatus and samples.

The detailed theory of IAC has been described elsewhere^4,8–11^. In short, the intensity of a photoelectron at the nth energy level is

(1)In(R→)∝|∑a,l,mCa,l,meik→⋅R→a(eiδl+1aXl+1,m+eiδl−1aXl−1,m)|2,

where *a* is the index of atom, *l* and *m* are the quantum numbers of atomic orbitals, *C*_*a*, *l*, *m*_ is the coefficient of atomic orbital *a*,*l*,*m*, *R*_*a*_ is the position vector of the *a*th atom, $\delta_{l\pm 1}^{a}$ is the overall phase shift of the final state, and *X*_*l*±1,*m*_ is the appropriate expression including radial matrix element of atomic transition from initial to final state^9^. *C*_*a*,*l*,*m*_ represents the share of the molecular orbital that is contributed by atomic orbital *a*, *l*, *m*; this share can be obtained from the result of DFT calculation. Although DFT is calculated for all of the atomic orbitals, we only use the orbits of the peripheral electrons (i.e., 2p in PAHs and 3p in BP). The contributions from core atomic orbitals are quite small and may be neglected for the valence band that is the region of interests^10^. The values of δ and the complete expression of X are listed in ref. 9.

### Computational settings

**Figure 1:**
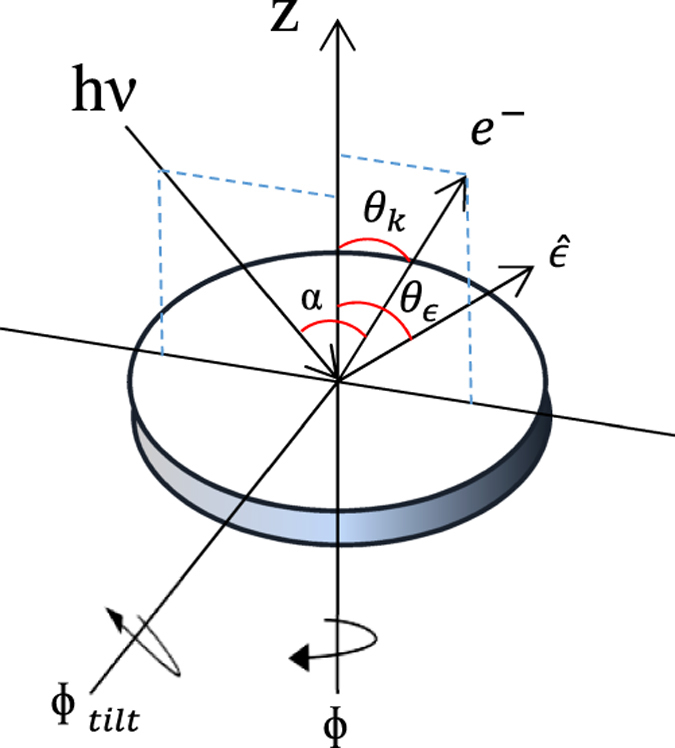
Geometry definition for experiment and simulation of ARPES. $\vec{k}$: direction of emitted photoelectron by incidence photon with polarization $\hat{\varepsilon }$. θ_k_: analyzer angle; ɸ: sample rotation angle; ɸ_tilt_: sample tilt angle.

The geometry of each angle is coded following the conventions suggested by Goldberg *et al*. (Figure 1) (ref. 9). For the calculation, we set the angle α between incidence X-ray and electron emission to 50° for comparison with experiments that were performed at the 8A2 beamline at the Pohang accelerator laboratory (PAL), Korea.

The first-principle results presented in this work were obtained using the Gaussian 09 package^13^. The structural geometry of molecules were optimized using the PBEh1PBE hybrid function with the sto-3g basis set^14^.

The simulations of photoelectron intensities using IAC calculation were performed by custom-made code. The calculation was done for every analyzer angle θ_k_ and sample rotation angle ɸ at a specific sample tilt angle ɸ_tilt_. For convenience, we set initial θ_k_=0 in the geometries of simulations and experiments.

### Experimental setting

ARPES experiments were conducted at the 8A2 HR-PES beam line of PAL using a high-resolution electron analyzer (VG Scienta SES 2002). The electron analyzer was normal from the sample surface and the incident angle of the p-polarized photon was 50°. The photon energies were varied from 100 to 400 eV. All experiments were performed at room temperature (RT). A ZYA-grade highly-oriented pyrolytic graphite (HOPG) (Materials Quartz Inc.) was cleaved using scotch tape outside an UHV chamber, then placed in the chamber immediately. The HOPG was degassed at 400 °C for >5 h to remove any adsorbed contaminants, then passively cooled to RT.

### Work flow

**Figure 2:**
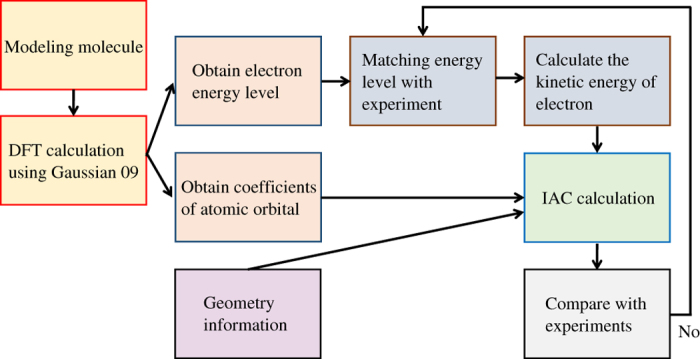
IAC calculation processing workflow for molecules.

Here, we describe the workflow (Figure 2) of the IAC calculation for a molecule. First, we modeled the atomic positions of a molecule in Cartesian coordinates. Then, using this modeled structure we performed DFT calculation including geometry optimization. The coefficients *C*_*a*,*l*,*m*_ were obtained from the output data of the Gaussian 09 package. Eigenvalues represent the energy level of molecular orbitals in units of Hartree. Because ARPES can analyze occupied states, we extracted every occupied energy level. To match the calculated energy level to that of the experiment, we scaled and shifted the calculated occupied energy level to match experimental energy levels of HOPG. This process was necessary because DFT calculation suffers from the approximation of electronic relaxation, and from correlation effects, so it cannot estimate energy levels to accurate absolute values.^15^ If comparable experimental data were not available, all energies were shifted so that the midpoint between highest occupied molecular orbital (HOMO) and lowest unoccupied molecular orbital (LUMO) was equivalent to zero binding energy.

For convenience, we output an _Inp file (Table 1) that tabulated *C*_*a*,*l*,*m*_ as a function of the kinetic energy of the emitted electron, the magnetic quantum number of the 2p or 3p orbital, and the atomic position. Radial matrix element and phase shift for a given photon energy are also included. The file format described (Table 1) is designed to calculate photoelectron intensity of every energy state.

**Figure 3:**
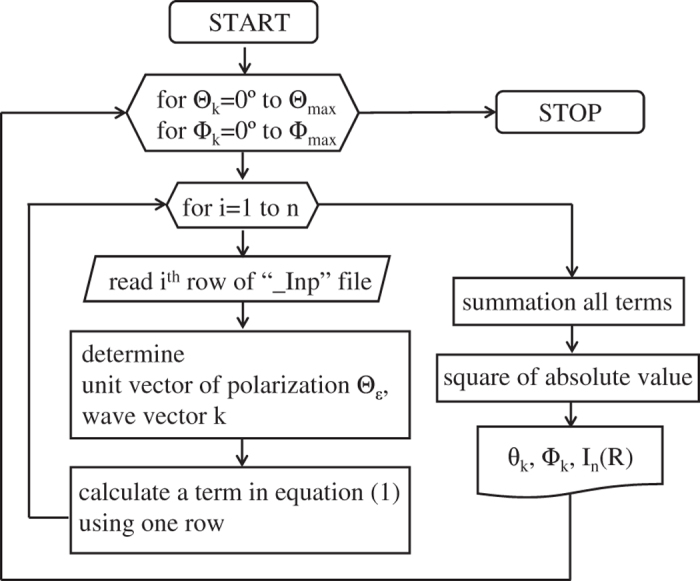
Algorithm flow chart for calculation using IAC approximation.

We used custom-made code that uses an algorithm (Figure 3) to calculate photoelectron intensity for every energy level. First, we read atomic coordinate, radial matrix element, phase shift, coefficient of atomic orbital, and kinetic energy of each molecular orbital from the _Inp file. Each row of _Inp file has information for the different independent atomic orbitals. The angles θ_k_ and ɸ_k_ of the detector can be selected to define the region of interest. The polarization θ_ε_ was determined from incidence angle θ_in_; the wave vector was determined from kinetic energy and detector angle. Then photoelectron intensity for one orbital can be calculated. By summing all terms for each molecular orbital then squaring the absolute value of the result, equation (1) was calculated with these variables by increasing θ_k_ and ɸ_k_. The calculated photoelectron intensity with IAC approximation was written to the _Result file as a function of θ_k_ and ɸ_k_ (Table 2). This process gives the photoelectron intensity as a function of analyzer position for a given sample and photon.

**Figure 4:**
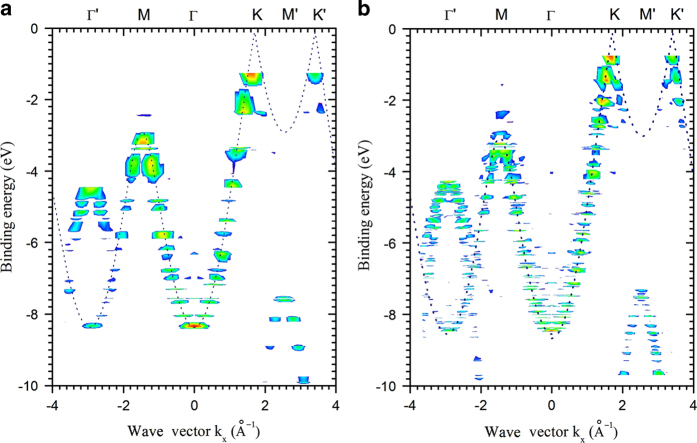
ARPES simulation for PAHs of K2 (**a**) and K4 (**b**) index with armchair edge. As the number of atom was increased, the resolution of the simulation was increased. Dotted lines: theoretical ɸ band structure of graphene. The negative wave vector points in the ΓM direction.

From the _Result file, we can simulate ARPES result as an image in k-space. The wave vector *k*_*⁄⁄*_ which is parallel to the sample surface can be calculated from the kinetic energy and emission angle as

(2)k⁄⁄=2mℏ2Ek⋅sinθk,

where the *E*_*k*_ is kinetic energy, which can be inferred from the occupied energy level, photon energy, and work function of the molecule. Then *k*_*⁄⁄*_ can be partitioned into vectors *k*_*x*_ and *k*_*y*_. Also, the result can be plotted with fixed ɸ as a function of binding energy to represent band structure; in PAHs ɸ is 0° in the ΓK direction and is 30° in the ΓM direction. As examples, ARPES simulations were conducted for PAHs of K2 and K4 index with armchair edge (Figure 4). The wave vectors of ΓK pointed in the positive direction and that of ΓM pointed in the negative direction. Also, we used every ɸ for a binding energy to represent an ARPES map of constant binding energy.

### Code availability

We used Labview to build home-made code that uses IAC approximation to calculate photoelectron intensity. The executable and source files can be downloaded from Dryad (Data Citation 1).

## Data Record

Numerical data that were generated using the Gaussian 09 package, custom-made simulation code, and experimental ARPES data of HOPG are available from Dryad (Data Citation 1). Data are given in tabular, tab-delimited value format.

To analyze the size effect of PAHs, we simulated ARPES of PAHs composed of 24 to 864 carbon atoms. The datasets for each simulation are classified distinctively (Table 3).

Each molecular simulation set consists of 11 subfiles (Table 4). They include calculation properties and atomic coordinates in _g09.com files and calculation results of Gaussian 09 in _g09.log files. The occupied energy level in _OccuEn.txt, kinetic energy of electron with 70-eV photon in _KE_Hv70.txt are listed in single columns. The _HOMO_constBEmap.txt file showing the PES intensity map of HOMO in k-space consists of *k*_x_, *k*_y_, and photoelectron intensity. The _gammaMband.txt and _gammaKband.txt files which contain band structure have three columns with wave vector, binding energy, and ARPES intensity in that order. The graphical representation of ARPES simulation for band structure is served as an _ARPES.jpg image file.

Experimental ARPES data files contain region 1 section and data section. The region 1 section specifies binding energy of each row in eV and angle of each column in degree. The data section stores photoelectron intensities in two dimensions (the first column shows binding energy).

## Technical Validation

The IAC theory for ARPES simulation of small molecules was well established and developed by Fadley^8,9^ and Ueno^11,16^. We followed the theory and used Gaussian 09 and custom-made code to extend the calculation to nano-scaled molecules. The custom-made code extracts every coefficient of every molecular orbital from the Gaussian 09 output file and uses equation (1) to calculate photoelectron intensity.

**Figure 5:**
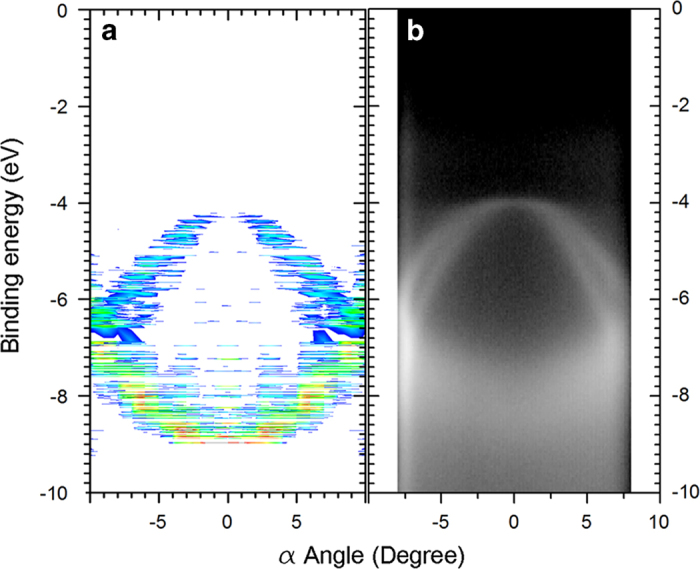
Comparison between simulation and experiment. (**a**) ARPES simulation of HOPG using IAC calculation. (**b**) ARPES experiment of HOPG at 8A2 beamline in PAL.

The results of ARPES simulation using IAC were validated by comparing them to the experimental results of HOPG (Figure 5). To simulate randomly-oriented HOPG, PES intensities of K6 PAH with armchair edge were averaged over the azimuthal angles ϕ. Specifically, the major agreements between the simulation and experiment show the same band structure that has overlapping σ 1 and σ 2 bands at the Γ point, and crossover of σ and π bands at higher angles. This result is comparable with graphene and graphite at the Γ point, because they have the same surface and band structure near the Γ point. We also reported simulations of 6°-tilted HOPG and BP and compared them with experimental data^4^.

## Usage Notes

We use Gaussian 09 package to obtain the coefficients of molecular orbitals but they may be obtained using other DFT calculation programs. Other datasets can be used by any software that can read data in txt (tab-separated value) for plotting and arranging.

## Additional Information

**How to cite**: Park, S. H. & Kwon, S. Modeling angle-resolved photoemission of graphene and black phosphorus nano structures. *Sci. Data* 3:160031 doi: 10.1038/sdata.2016.31 (2016).

## Supplementary Material



## Figures and Tables

**Table 1:** Format of _Inp file with several parameters for the IAC calculation.

**Filename suffix**	**Filename extension**	**Description**
_img	Tif	atomic image of molecule to calculate.
_g09	Com	input file of DFT calculation (Gaussian09 package) for head and atomic position.
_g09	Log	DFT calculation result file containing energy level and LCAO.
_OccEn	Txt	Molecule energy level of occupied states evaluated from log file.
_KE_Hv	Txt	kinetic energy of emitted electron at each occupied state for given photon energy and work function.
_Inp_Hv	Txt	table for calculating photoelectron intensities. See table 1 for detail.
_Result_Hv_ThIn	Txt	calculated photoelectron intensities as a function of ɸ and θ for given geometry. See table 2 for detail.
_HOMO_constBE map	Txt	photoelectron intensities for constant binding energy at HOMO level in momentum space.
_GammaMband	Txt	photoelectron intensities showing band structure along ΓM direction.
_GammaKband	Txt	photoelectron intensities showing band structure along ΓK direction.
_ARPES	Jpg	graphical representation of ARPES simulation along the ΓK and ΓM direction.

**Table 2:** Format of _Result file tabulate the result of the IAC calculation. The ARPES intensity are written for ɸ and *θ*_k_. The kinetic energy of each column is matched with that of _Inp file (Table 1).

0	0	0	0	0	0	0	0	0	46	47	•••
0	−1	2.2	1	0	0.1	0.2	2.7	1.3	−0.01	−0.07	•••
0	1	2.2	1	0	0.1	0.2	2.7	1.3	−0.02	-0.08	•••
0	0	2.2	1	0	0.1	0.2	2.7	1.3	0	0	•••
Dummy	Magnetic quantum number								Atomic position			Radial matrix element^9^		Phase shift^9^		Kinetic energy of electron at each state		
	2p for PAH 3p for BP	X	Y	Z	Rs	Rd	Ds	Dd	Coefficient, C_a,l,m_					

**Table 3:** Classifying simulation set of PAHs by size and edge structure in addition of BP.

ɸ	**θ**_**k**_	**ARPES intensity for the kinetic energy of _inp file**			
0	0	0.00038	0.00162	0.00202	•••
90	0	0.00385	0.01277	0.01573	•••
180	0	0.00019	0.00054	0.00063	•••
270	0	0.00204	0.00544	0.00605	•••
0	2	0.00249	0.01254	0.00023	•••
•	•	•	•	•	•
•	•	•	•	•	•
•	•	•	•	•	•

**Table 4:** Subfiles and their descriptions for ARPES simulation of each molecule.

**Filename**	**Sample**	**Index K**	**Number of C atoms**	**Edge structure**	**Description**
K1AMC	PAH	1	42	Armchair	ARPES simulation set for 42 C-atom PAHs with armchair edge
K2AMC	PAH	2	114	Armchair	ARPES simulation set for 114 C-atom PAHs with armchair edge
K3AMC	PAH	3	222	Armchair	ARPES simulation set for 222 C-atom PAHs with armchair edge
K4AMC	PAH	4	366	Armchair	ARPES simulation set for 366 C-atom PAHs with armchair edge
K6AMC	PAH	6	762	Armchair	ARPES simulation set for 762 C-atom PAHs with armchair edge
HOPG	PAH	6	762	Armchair	ARPES simulation set for K6AMC averaged over ɸ
HOPGtilt	PAH	6	762	Armchair	ARPES simulation set for HOPG with ɸ_tilt_ = 6°
K1ZIG	PAH	1	24	Zigzag	ARPES simulation set for 24 C-atom PAHs with zigzag edge
K3ZIG	PAH	3	96	Zigzag	ARPES simulation set for 96 C-atom PAHs with zigzag edge
K5ZIG	PAH	5	216	Zigzag	ARPES simulation set for 216 C-atom PAHs with zigzag edge
K7ZIG	PAH	7	384	Zigzag	ARPES simulation set for 384 C-atom PAHs with zigzag edge
K11ZIG	PAH	11	864	Zigzag	ARPES simulation set for 864 C-atom PAHs with zigzag edge
BP	BP	‘	278 (P)	‘	ARPES simulation set for 278 atoms of black phosphorus
